# High prevalence of erectile dysfunction in men with hyperthyroidism: a meta-analysis

**DOI:** 10.1186/s12902-024-01585-6

**Published:** 2024-04-30

**Authors:** Xiaowen Liu, Yanling Wang, Li Ma, Danhui Wang, Zhihong Peng, Zenghui Mao

**Affiliations:** 1 Hunan Provincial Key Laboratory of Regional Hereditary Birth Defects Prevention and Control, Changsha Hospital for Maternal & Child Health Care, Hunan Normal University, Changsha, China; 2 College of Health Science and Engineering, Hubei University, Wuhan, China

**Keywords:** Erectile dysfunction, Hyperthyroidism, Morbidity, Therapy

## Abstract

**Objective:**

The objective of this study was to evaluate the association between hyperthyroidism and the risk of developing erectile dysfunction (ED).

**Methods:**

A comprehensive search of multiple databases, including PubMed, Embase, Cochrane, and Web of Science, was conducted to identify relevant studies investigating the relationship between hyperthyroidism and ED in men. The quality of the included studies was assessed using the Newcastle‒Ottawa Quality Rating Scale, and a meta-analysis was performed using Stata 16.0 and RevMan 5.3 software.

**Results:**

A total of four papers encompassing 25,519 study subjects were included in the analysis. Among these, 6,429 individuals had hyperthyroidism, while 19,090 served as controls. The overall prevalence of ED in patients with hyperthyroidism was determined to be 31.1% (95% CI 0.06–0.56). In patients with uncomplicated hyperthyroidism, the incidence of ED was 21.9% (95% CI 0.05–0.38). The combined odds ratio (OR) for the four studies was 1.73 (OR: 1.73; 95% CI [1.46–2.04]; *p* < .00001).

**Conclusion:**

Our findings demonstrate a higher incidence of ED in patients with hyperthyroidism. These results provide valuable information for healthcare professionals and can facilitate discussions surrounding appropriate treatment options for ED in patients with hyperthyroidism.

**Supplementary Information:**

The online version contains supplementary material available at 10.1186/s12902-024-01585-6.

## Introduction

Erectile dysfunction (ED), a prevalent condition globally, is characterized by the inability of men to achieve or sustain an erection sufficient for satisfactory sexual performance. This condition is typically persistent for a duration of more than three months [1, 2]. As a chronic ailment, ED significantly impacts physical and mental well-being, as well as overall quality of life, for both affected men and their partners. Research suggests that there is a need for screening and risk assessment for men with ED, rather than solely focusing on treatment options [3]. However, the precise underlying risk factors contributing to ED remain unclear.

Hyperthyroidism characterized by the excessive secretion of thyroid hormones from the thyroid gland, leads to hypermetabolism and increased excitability of the circulatory, digestive, and nervous systems [4]. Research indicates that individuals with hyperthyroidism are more susceptible to conditions such as anxiety, depression, and affective disorders than the general population [5–7]. The incidence of hyperthyroidism has been reported to be 0.816% in Norway, 0.276% in Sweden, and approximately 1.25% in China [8, 9]. However, the association between hyperthyroidism and the occurrence of erectile dysfunction (ED) remains uncertain.

The objective of this study was to investigate the potential impact of hyperthyroidism on erectile dysfunction (ED) in men. By examining this association, we aimed to provide new insights that could inform the treatment approach for male patients with ED.

## Materials and methods

This systematic review complies with the PRISMA 2009 (Preferred Reporting Items for Systematic Reviews and Meta-analyses) statement.

### Data sources and search strategies

All studies evaluating men with ED suffering from hyperthyroidism were obtained by searching the PubMed, Embase, Cochrane, and Web of Science databases. The search was conducted from the date of database creation to March 26, 2024. Briefly, the search keywords were (Hyperthyroid OR Primar Hyperthyroidism) AND (Dysfunction, Erectile OR Male Impotence), and only literature with English as the language of publication was collected for all results.

### Study selection

#### Inclusion criteria:

1. study subjects aged ≥ 18;
2. use of a validated tool for diagnosing ED, e.g., the International Index of Erectile Function (IIEF-5) and SHIM questionnaire;
3. All subjects had their TSH hormone expression levels (mU/liter) measured in the body.
4. All subjects underwent assessment of intrabody expression levels of TSH hormone and male erectile function to explore the correlation between the two. Additionally, RR (relative risk) values and OR values were provided.

#### Exclusion criteria:

1. Study subjects aged < 18;
2. Duplicate published literature, review literature, animal experimental literature, etc.;
3. literature for which the needed data were not available and the authors could not be contacted;
4. literature for which the needed study data were not available, such as: RR or OR.

### Data extraction and Quality assessment

**Fig. 1:**
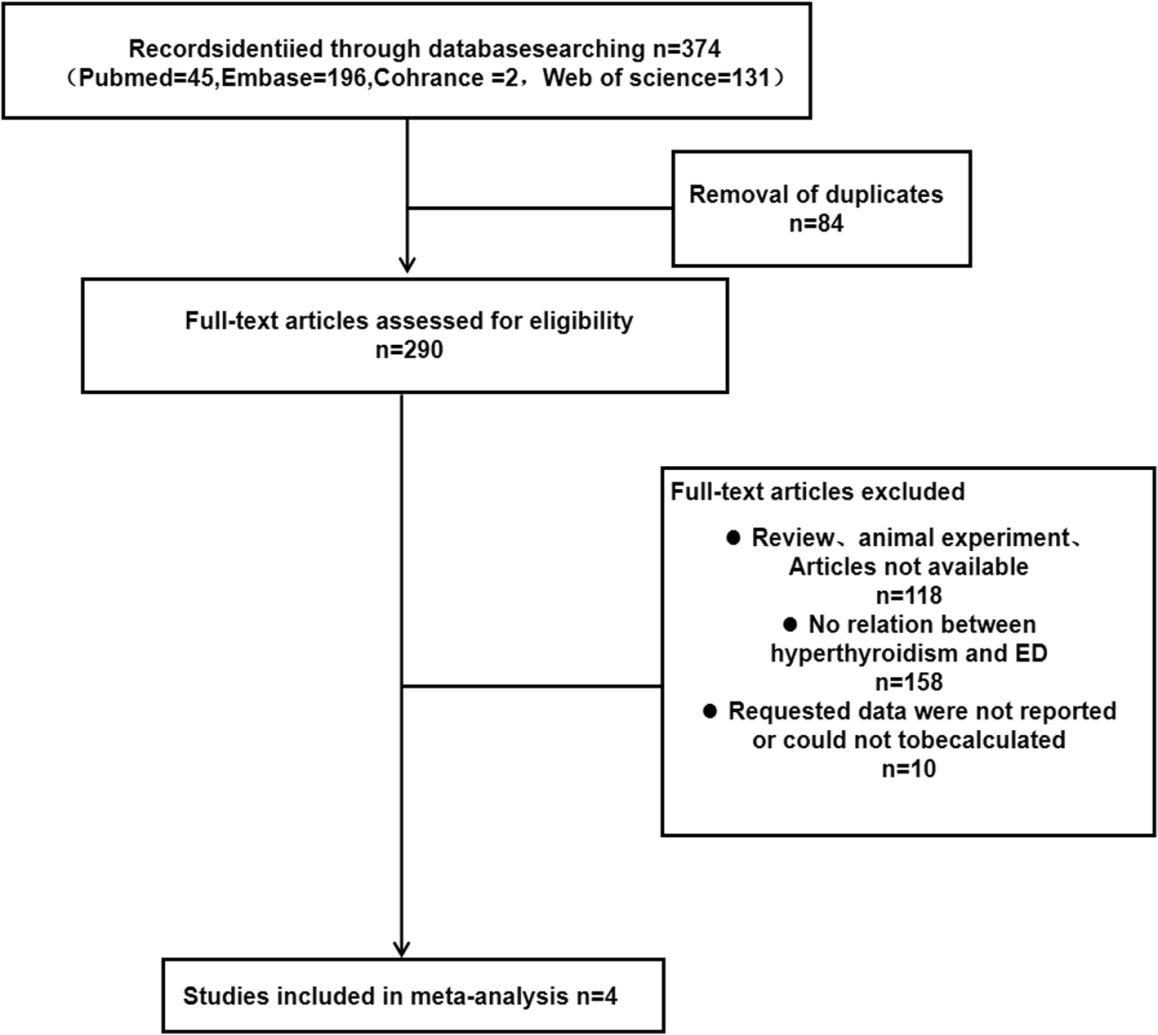
Study selection. Literature search for the meta-analysis

Two reviewers, X. L. and Y. L. W., conducted an independent screening of titles and abstracts. Full-text articles were obtained for further evaluation based on the initial screening (Fig. 1). The search strategy was developed collaboratively by an experienced librarian and two investigators. All relevant data from the selected studies were extracted and recorded using a standardized approach. In cases where differences of opinion arose, consensus was reached through discussion. The extracted data included information on the author, publication year, country of origin, method of ED measurement, and sample size of the hyperthyroidism and control groups. The final included literature underwent quality assessment using the Newcastle‒Ottawa Scale (NOS) by the researchers.

### Data synthesis

Meta-analysis was performed using Review Manager 5.3 and Stata 16 software, and by software heterogeneity test, when *P*>0.1 and *I*^*2*^<50%, it suggested good homogeneity of results among studies, and the fixed-effect model was used to combine OR values; conversely, if *P*≤0.1 and/or *I*^*2*^≥50%, it suggested heterogeneity of results among studies, and further analysis of the reasons for the existence of heterogeneity was needed, and after excluding the obvious. After excluding the effects of obvious clinical heterogeneity and methodological heterogeneity, if the heterogeneity was still relatively large, a random-effects model or a descriptive analysis was used. Publication bias was assessed using funnel plots, Begg's test, and Egger's test.

## Result

### Literature search and included studies

Our literature search identified 374 titles and abstracts, which yielded four articles potentially relevant for inclusion after screening (Fig. 1).

### Study and patient characteristics

We included 4 cohort studies (6429 patients out of 25519 people) published and appraised using the Newcastle‒Ottawa Scale (NOS). Overall, the studies were of good methodological quality and were rated as ≥6 across most items. All findings are displayed in Table 1 [10–13].

**Table 1:** Summary of studies included in the meta-analysis

First author	Country	Age(mean ± SD)	No. of Ht patients with/without	No. of non-Ht patients with/without	ED measure	Study quality	TSF level in patients with hyperthyroidism (mU/liter)
Gerasimos E, 2008 [10]	Greece	52.6 ± 13.7	27/45	24/47	SHIM	7	< 0.3
J Keller, 2012 [11]	China	18–79	207/6103	362/18568	IIEF-5	6	< 0.05
Cesare Carani, 2005 [12]	Italy	43.2 ± 12	5/29	1/33	IIEF-5	6	< 0.1
A Veronelli, 2006 [13]	Italy	36–78	10/3	30/25	IIEF-5	6	0.01–3.06

*SHIM *Sexual Health Inventory for Men, *IIEF-5* International Index of Erectile Function-5, *Ht *hyperthyroidism, *TSH* thyroid stimulating hormone

### Statistical analysis results

**Fig. 2:**
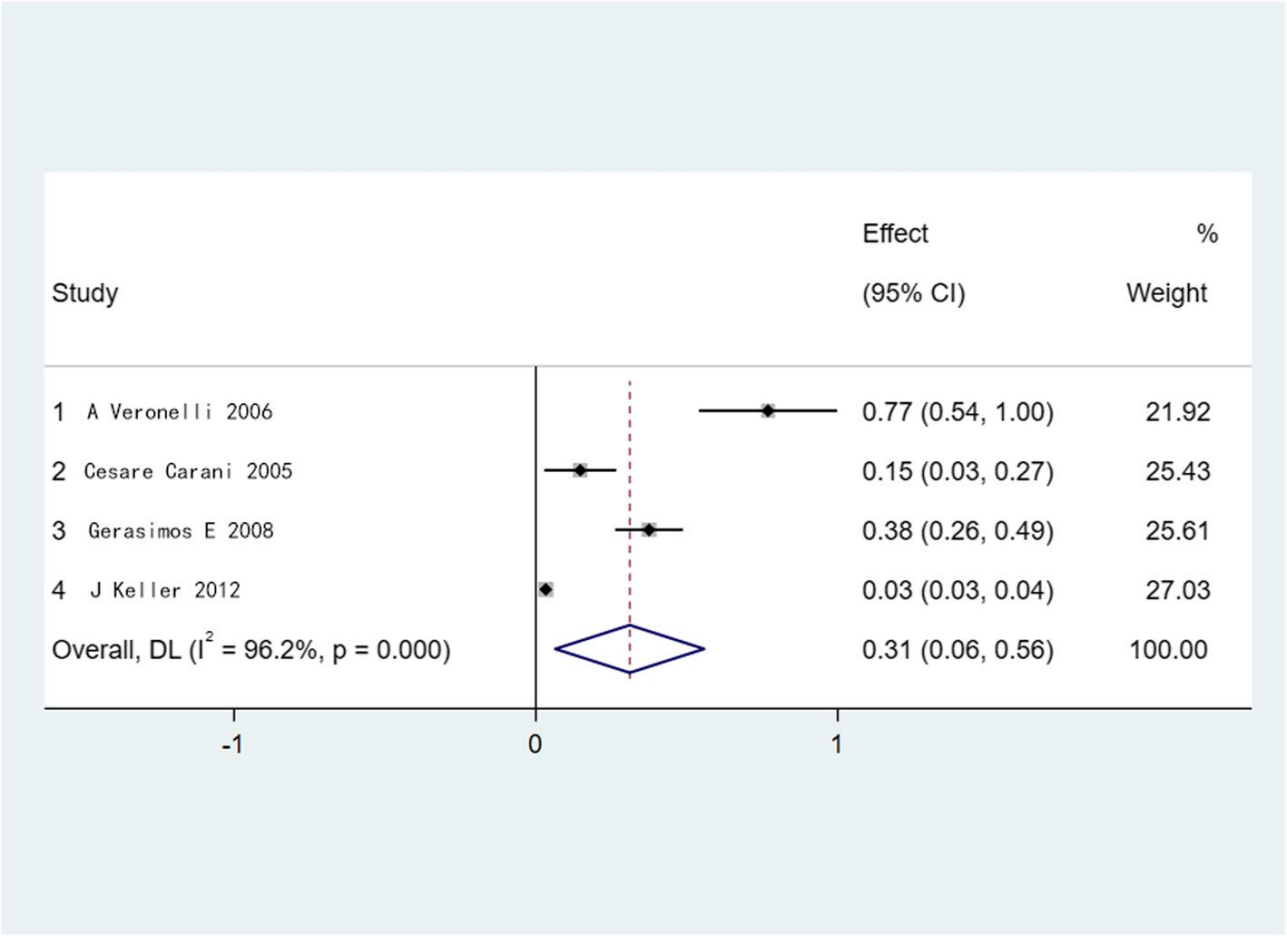
Meta-analysis of the prevalence of ED in patients with comorbid hyperthyroidism. Heterogeneity, *I*^*2*^*;* effect size, Effect; Confidence intervals, CI

**Fig. 3:**
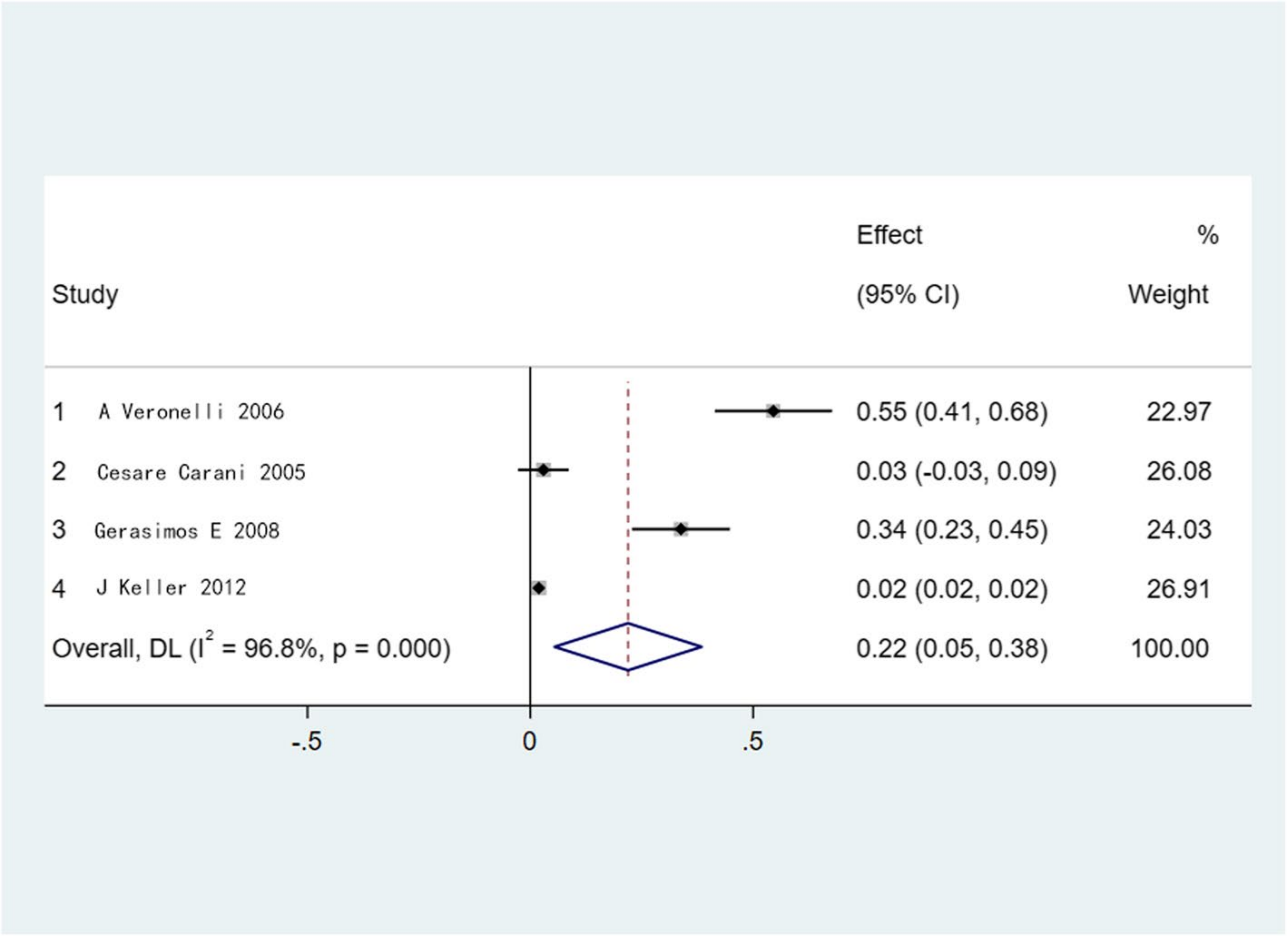
Meta-analysis of the prevalence of ED in patients with combined uncomplicated hyperthyroidism. Heterogeneity, *I*^*2*^*;* effect size, Effect; Confidence intervals, CI

Four studies were included in the analysis, and the results showed that the overall prevalence of ED in patients with comorbid hyperthyroidism was 31.1% (95% CI 0.06-0.56) (Fig. 2). The overall prevalence of ED in patients with combined uncomplicated hyperthyroidism was 21.9% (95% CI 0.05-0.38) (Fig. 3). Therefore, these results suggested that patients with hyperthyroidism had a higher incidence of ED. Given the relationship between TSH levels and ED, as well as the observed heterogeneity among groups, we conducted a subgroup analysis (Supplementary figures 1 and 2). The subgroup analysis revealed that the differences in TSH levels across the studies were not the source of heterogeneity.

### The association of ED and hyperthyroidism

**Fig. 4:**
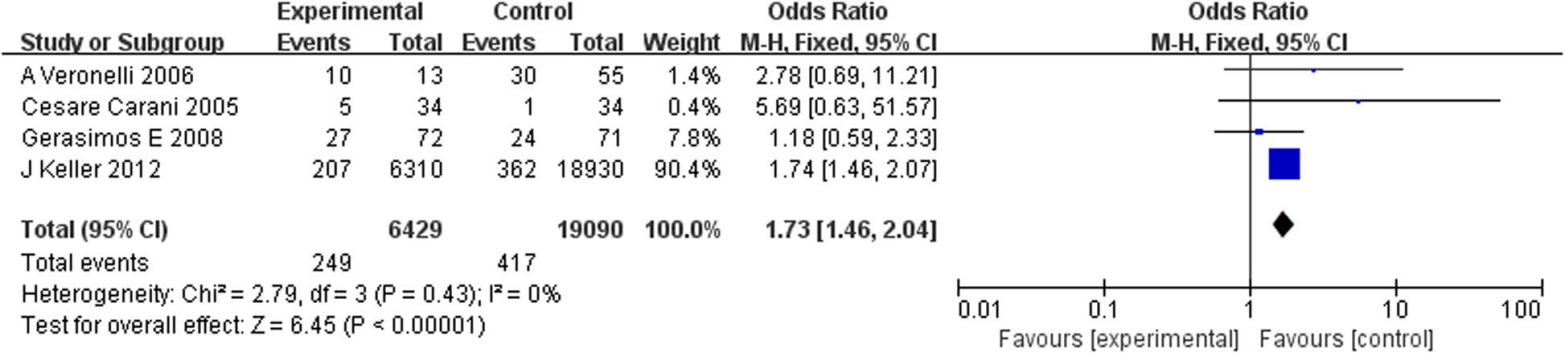
Meta-analysis of crude OR for ED compared with non-ED. Heterogeneity, *I*^*2*^*;* effect size, Effect; Confidence intervals, CI

**Fig. 5:**
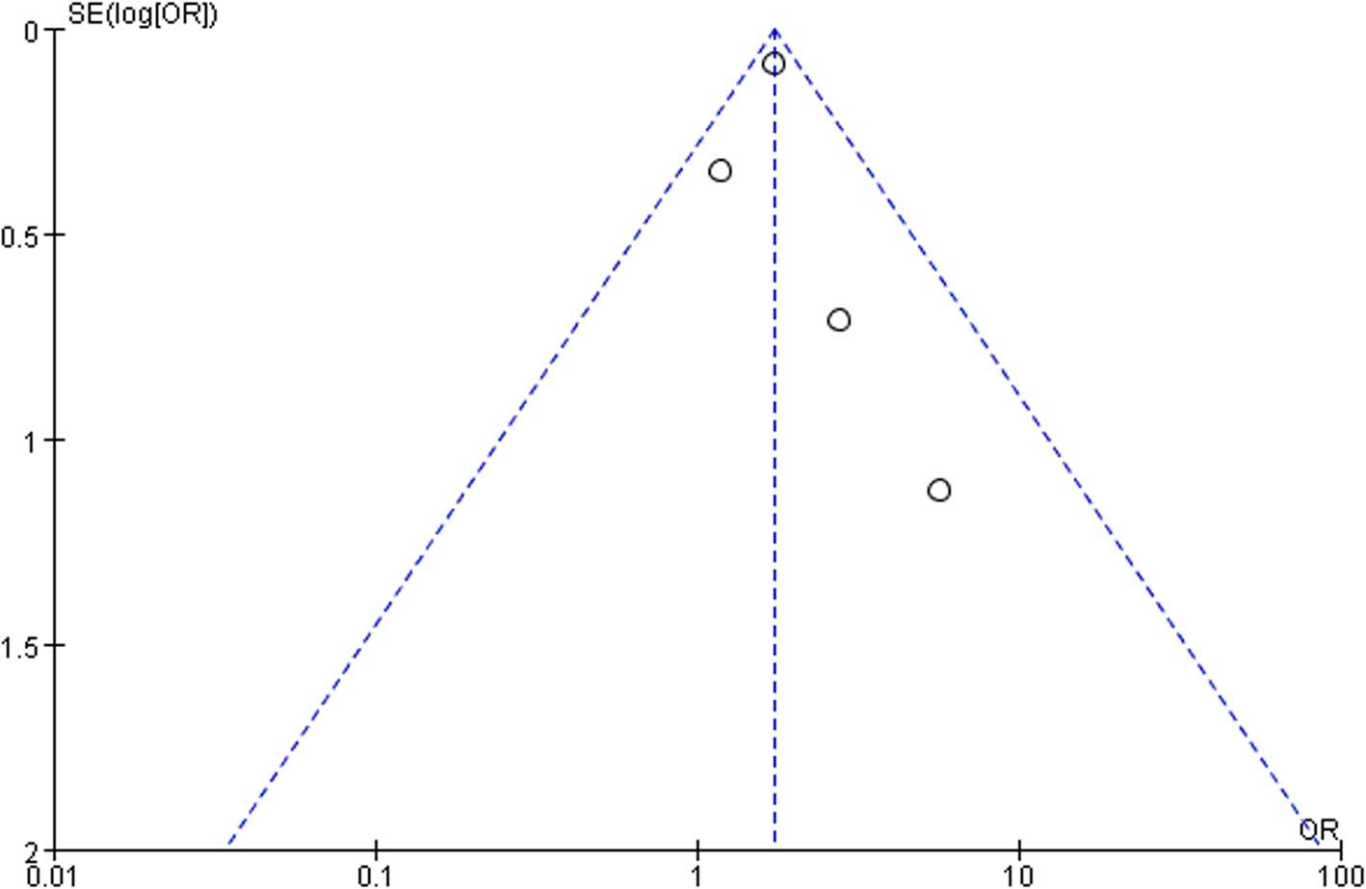
Funnel plot of publication bias. The funnel plot uses the OR as the x-axis and the reciprocal of the standard error of the log odds ratio (1/SE[logOR]) as the y-axis

The OR (odds ratio) was calculated based on the number of hyperthyroid patients with ED, hyperthyroid patients without ED, nonhyperthyroid patients with ED, and nonhyperthyroid patients without ED, and the results showed a ratio of 1.73 (95% CI 1.46-2.04) for the four studies combined and *P*<0.00001 with *I*^*2*^≤50% (Fig. 4), indicating that there was no heterogeneity between the study results, so a fixed-effects model was conducted. Furthermore, no publication bias was found by Begg's test (*P* = 0.065), Egger's test (*P* = 0.065), or funnel plot analysis (Fig. 5).

## Discussion

This meta-analysis involved the compilation of clinical case studies focused on patients with both erectile dysfunction (ED) and hyperthyroidism. In total, four papers encompassing a population of 25,519 study subjects were included in the analysis. Among these subjects, there were 6,429 patients in the hyperthyroidism group and 19,090 in the control group. The overall quality score of the included literature, as assessed using the NOS, was deemed to be good. Significant differences were observed in all study metrics between the hyperthyroidism and control groups (*p* < 0.00001). To assess publication bias, Begg's test (*p* = 0.065) and Egger's test (*p* = 0.065), along with funnel plots, were employed, demonstrating the absence of such bias. The findings of our analysis indicated that the prevalence of ED in men with hyperthyroidism was 1.73 times higher than that in men without hyperthyroidism. Considering the well-established relationship between thyroid-stimulating hormone (TSH) and ED, we conducted a subgroup analysis based on different TSH levels to investigate potential sources of heterogeneity. However, the results of this analysis indicated that the variance in TSH levels did not contribute to the observed heterogeneity.

Thyroid hormones are essential for normal human development and play a crucial role in maintaining male fertility. The secretion of thyroid hormones has been found to impact male reproductive function at both the microscopic and macroscopic levels. For instance, thyroid hormone levels can influence intratesticular spermatogenesis, semen quality, and sex hormone levels [14]. The two main disorders associated with abnormal thyroid hormone secretion are hyperthyroidism and hypothyroidism. Overt hypothyroidism has been shown to cause significant sexual dysfunction (SD) in both men and women, although the correlation between subclinical hypothyroidism and sexual dysfunction is not clearly established [15]. Thyroid disease is strongly linked to ejaculatory dysfunction in men, and it has been observed that hypothyroid patients have a higher incidence of delayed ejaculation than hyperthyroid patients [8]. In studies investigating the relationship between male erectile function and thyroid hormones, researchers have emphasized the important role of thyroid hormones in the erectile process. However, the precise pathophysiology of sexual dysfunction linked to thyroid activity is still not fully understood. Furthermore, researchers have noted that as men age, there tends to be a decline in overall and sexual health, which is closely associated with the continuous decrease in testosterone levels [16]. A recent paper aligns closely with our perspective, asserting that there is a significant correlation between hyperthyroidism and sexual function, affecting not only men but also women [17].

Erectile dysfunction (ED) is a prevalent male sexual function disorder that commonly affects men over the age of 40. The global prevalence of ED varies between 3% and 76.5%, and projections suggest that by 2025, approximately 322 million men worldwide may experience ED [18, 19]. The etiology of ED is multifactorial and complex. Age, diabetes, dyslipidemia, hypertension, cardiovascular disease (CVD), body mass index (BMI), obesity, waist circumference, metabolic syndrome (MetS), hyperhomocysteinemia, sedentary lifestyle, smoking, and certain medications, such as thiazide diuretics, are recognized as common risk factors for ED [20–23]. Furthermore, various medical conditions have been associated with ED, including atrial fibrillation, hyperthyroidism, vitamin D deficiency, and chronic obstructive pulmonary disease (COPD) [24–27]. Thyroid hormones and testosterone play crucial roles in metabolism, growth, development, and immune regulation. They act as key regulators of energy metabolism, and research suggests that they promote the transcription of genes related to oxidative phosphorylation (OXPHOS) and the biosynthesis of OXPHOS within mitochondria [28]. Thyroid hormones directly impact the development and metabolism of testicular and cavernous tissues through specific receptors [29, 30]. Additionally, an increase in thyroid hormone concentration enhances liver metabolism and hepatic nuclear factor-4α levels, thereby stimulating the promoter of sex hormone-binding globulin (SHBG) and increasing its transcription and concentration [31]. As SHBG has a higher affinity for testosterone than for estradiol, thyroid dysfunction may disrupt the balance of available sex hormones in males, potentially leading to erectile dysfunction.

In recent years, there has been growing evidence suggesting that erectile dysfunction (ED) is not only prevalent in hyperthyroid patients but also has a significant impact on their quality of life [32]. Although there is no direct association between hyperthyroidism and ED, the combination of hyperthyroidism with other diseases, such as diabetes mellitus (DM) and depression, can further increase the prevalence of ED. Several studies have shown that the prevalence of DM in men with ED is 19.7%, and the prevalence of depression is 11.9% [33]. Additionally, the prevalence of ED in men with diabetes mellitus and metabolic syndrome (DS) was found to be approximately twice that of men with diabetes mellitus alone [34]. It is important to note that the occurrence of ED can also influence the development of both DM and DS, creating a cyclical relationship. While current treatment options can effectively manage ED, they do not offer a cure for the condition, except in specific cases such as psychogenic ED, posttraumatic arteriogenic ED in younger patients, and hormone-related ED [35, 36]. These cases may benefit from targeted treatments addressing the underlying causes. The most commonly used treatments for ED are oral phosphodiesterase type 5 inhibitors (PDE5Is), such as sildenafil, tadalafil, vardenafil, and avanafil. These drugs increase nitric oxide levels in blood vessels, improving penile blood flow, and are considered the first-line treatment for ED [37]. Another option is the use of topical treatments, such as prostaglandin E1 (Alprostadil), which can be applied either topically or intraurethrally. These medications work by directly relaxing penile smooth muscles and increasing blood flow [38]. Low-intensity shock wave therapy (LI-SWT) is a noninvasive therapy that stimulates the formation of new blood vessels, thereby improving penile blood flow [39]. Other therapies for ED include vacuum erection devices (VEDs), intracavernosal injection therapy, penile implants, psychological counseling, and lifestyle modifications [40–44]. The choice and application of these methods depend on individual circumstances, and there is no universal treatment suitable for all patients or situations. It is essential to consult with a healthcare professional to determine the most appropriate treatment for each individual case, considering their underlying conditions and overall health.

The limitations of this study warrant careful consideration. First and foremost, the existing body of published literature focusing on clinical data related to hyperthyroidism and ED is limited, necessitating further research to validate the findings presented herein. Secondly, the collection of additional outcome indicators, such as thyroid-stimulating hormone, serum T3, and serum T4, is limited. It is hoped that future studies will include a broader range of clinical observation indicators to further substantiate the results of the experiments.

## Conclusion

Understanding the relationship between erectile dysfunction (ED) and hyperthyroidism is crucial for both clinical male infertility specialists and hematologists in making appropriate diagnostic and therapeutic decisions. This meta-analysis indicates a higher incidence of ED in patients with hyperthyroidism, suggesting a potential association between the two conditions. However, further research is needed to determine whether lower blood markers, such as thyroid stimulating hormone (TSH), T3, and T4 values, are independent risk factors for ED. Investigating this relationship will provide valuable insights into the mechanisms underlying the association and help guide clinical decisions. Additionally, it is important to determine whether lower blood markers in hyperthyroid patients contraindicate the use of antithyroid drugs. Antithyroid drugs are commonly used to treat hyperthyroidism, but their potential impact on ED needs to be evaluated. Future studies should focus on assessing whether the use of these drugs exacerbates or improves ED symptoms in patients with hyperthyroidism. By conducting further research, healthcare professionals specializing in male infertility and hematology can gain a better understanding of the relationship between ED and hyperthyroidism. This knowledge will aid in the development of more tailored diagnostic approaches and treatment strategies, enhancing patient care in both fields.

### Supplementary Information

**Supplementary Material 1.**

**Supplementary Material 2.**

## Data Availability

The datasets used and/or analyzed during the current study are available from the corresponding author upon reasonable request.
