# Supplementary material for: High prevalence of erectile dysfunction in men with hyperthyroidism: a meta-analysis
Source: BMC Endocr Disord. 2024 Apr 30;24:58. doi: 10.1186/s12902-024-01585-6 (PMC11059661; doi:10.1186/s12902-024-01585-6)
Supplement: Supplementary file 1 — Supplementary Material 1. [file 12902_2024_1585_MOESM1_ESM.pdf]

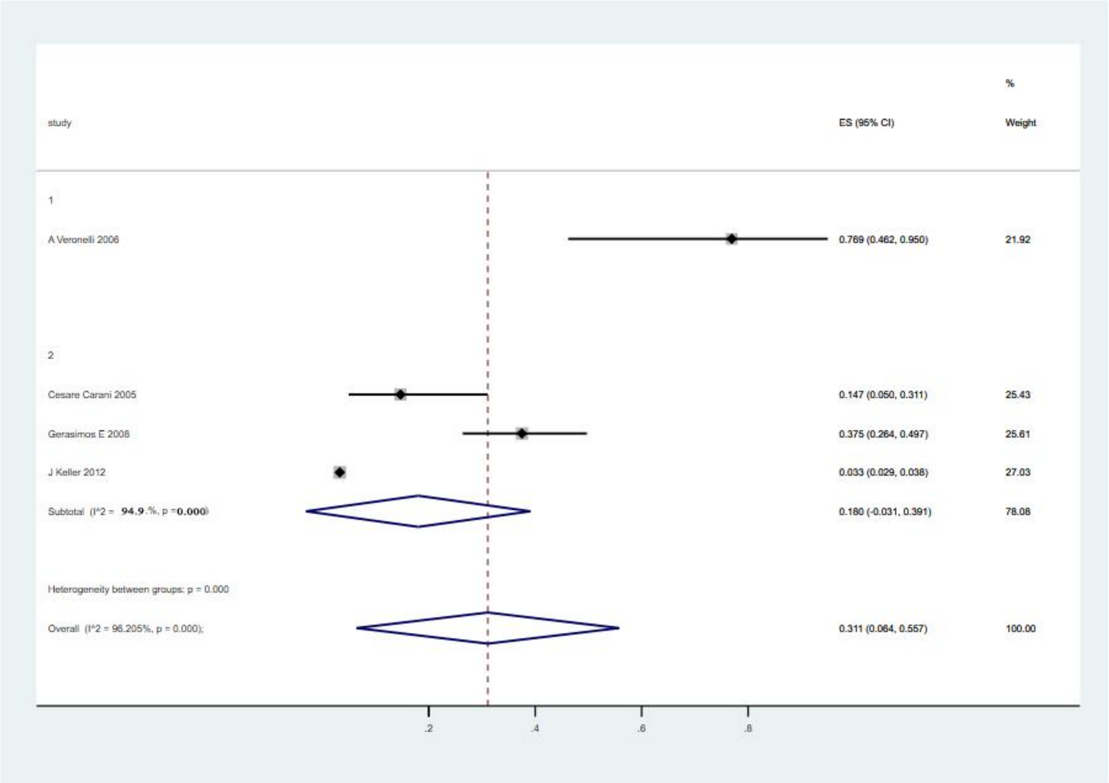

supplementary figure 1:Utilizing subgroup analysis to determine the sources of heterogeneity. Conduct subgroup analysis based on different TSH levels to detect the heterogeneity of ED prevalence among patients with concomitant hyperthyroidism. Heterogeneity, I^2; effect size, Effect; Confidence intervals, CI.
