# Supplementary material for: High prevalence of erectile dysfunction in men with hyperthyroidism: a meta-analysis
Source: BMC Endocr Disord. 2024 Apr 30;24:58. doi: 10.1186/s12902-024-01585-6 (PMC11059661; doi:10.1186/s12902-024-01585-6)
Supplement: Supplementary file 2 — Supplementary Material 2. [file 12902_2024_1585_MOESM2_ESM.pdf]

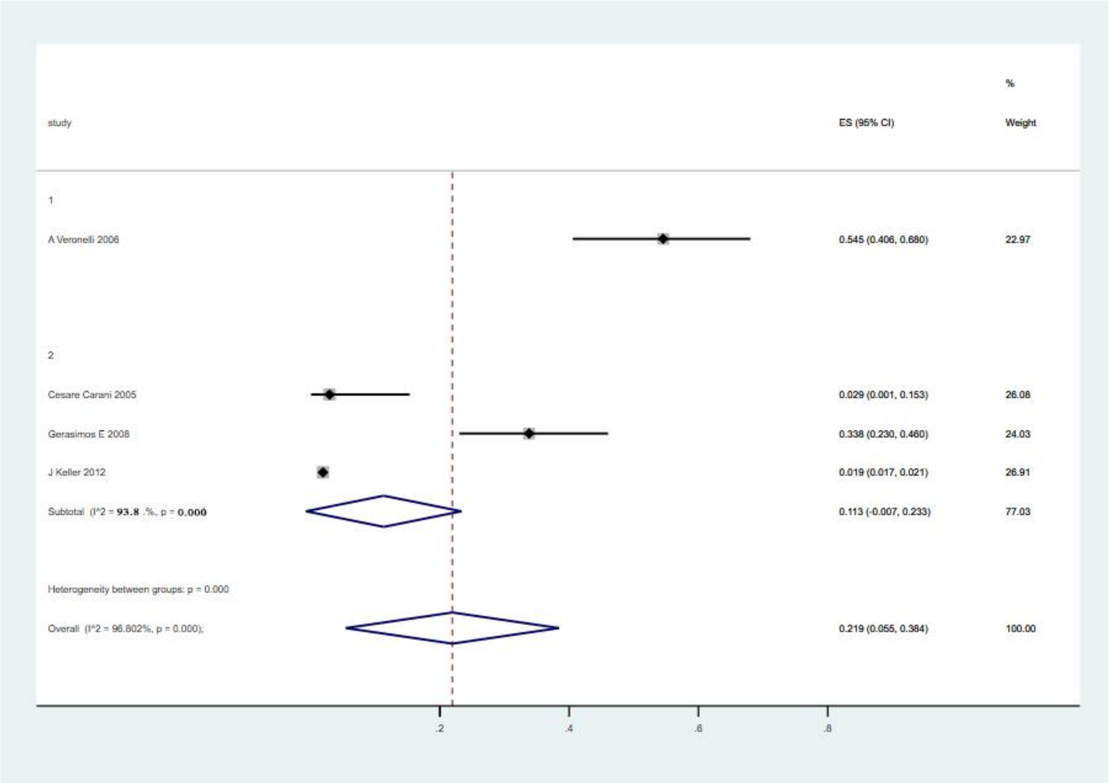

supplementary figure 2:Utilizing subgroup analysis to determine the sources of heterogeneity. Conduct subgroup analysis based on different TSH levels to detect the heterogeneity of ED prevalence among patients with combined uncomplicated hyperthyroidism. Heterogeneity, I^2; effect size, Effect; Confidence intervals, CI.
